# Correction to: Ultra-low-field brain MRI morphometry: Test–retest reliability and correspondence to high-field MRI

**DOI:** 10.1162/IMAG.x.1261

**Published:** 2026-05-26

**Authors:** František Váša, Carly Bennallick, Niall J. Bourke, Francesco Padormo, Levente Baljer, Ula Briski, Paul Cawley, Tomoki Arichi, Tobias C. Wood, David J. Lythgoe, Flavio Dell’Acqua, Thomas C. Booth, Ashwin V. Venkataraman, Emil Ljungberg, Sean C.L. Deoni, Rosalyn J. Moran, Robert Leech, Steven C.R. Williams

**Affiliations:** Department of Neuroimaging, IoPPN, King’s College London, London, United Kingdom; Hyperfine, Inc., Guilford, CT, United States; Research Department of Early Life Imaging, School of Biomedical Engineering and Imaging Sciences, King’s College London, London, United Kingdom; Medical Research Council Center for Neurodevelopmental Disorders, King’s College London, London, United Kingdom; Department of Forensic and Neurodevelopmental Sciences, IoPPN, King’s College London, London, United Kingdom; School of Biomedical Engineering & Imaging Sciences, King’s College London, London, United Kingdom; Department of Neuroradiology, Ruskin Wing, King’s College Hospital NHS Foundation Trust, London, United Kingdom; Centre for Healthy Brain Ageing, IoPPN, King’s College London, London, United Kingdom; Department of Medical Radiation Physics, Lund University, Lund, Sweden; Bill and Melinda Gates Foundation, Seattle, WA, United States

In the original manuscript, there were two issues with the application of the multi-resolution registration (MRR) step of our analysis, applied to combine three orthogonal ultra-low-field acquisitions with low through-plane resolution (AXI, COR, SAG) into a single higher-resolution scan.

First, due to an error in our code (pointed out following its release on GitHub), our MRR analysis involved combination of COR and SAG scans only (i.e., the AXI scan was not included in the *ANTs* command which is central to executing MRR). Second, to align inputs before running MRR, we pre-registered each ultra-low-field (64 mT) scan to the participant’s corresponding high-field (3 T) scan; however, this raises concerns regarding circular use of high-field data, which is subsequently used as a reference standard.

We now repeated all analyses including the AXI scan, using two different pre-registration approaches: the original pre-registration to individual high-field scans, and an alternative pre-registration to a standard high-field template (MNI ICBM152 nonlinear asymmetric), of the same contrast as the input ultra-low-field scans.

The impact on results was minor, and is summarised within the following table (an extension of Table 1), depicting summary statistics capturing both 64 mT test–retest reliability, and correspondence to 3 T.

**Table 1.** Summary of regional reliability and correspondence, across contrasts, for the original multi-resolution registration (MRR) approach and two corrected approaches.

	Contrast	MRR (HF, 2-plane)	MRR (HF, 3-plane)	MRR (MNI, 3-plane)
ICC	T_1_w	0.96 [0.93,0.98]	0.98 [0.95,0.99]	0.97 [0.94,0.98]
	T_2_w	0.97 [0.93,0.98]	0.97 [0.95,0.98]	0.97 [0.95,0.98]
Md Dice_rel_	T_1_w	0.79 [0.77,0.82]	0.81 [0.79,0.83]	0.80 [0.78,0.82]
	T_2_w	0.81 [0.79,0.84]	0.81 [0.79,0.84]	0.81 [0.79,0.83]
Pearson’s r	T_1_w	0.93 [0.87,0.95]	0.93 [0.88,0.96]	0.93 [0.88,0.95]
	T_2_w	0.94 [0.90,0.96]	0.95 [0.92,0.96]	0.93 [0.89,0.96]
Lin’s CCC	T_1_w	0.60 [0.45,0.72]	0.53 [0.39,0.67]	0.52 [0.32,0.72]
	T_2_w	0.89 [0.83,0.93]	0.90 [0.83,0.94]	0.85 [0.73,0.92]
Md Dice_cor_	T_1_w	0.61 [0.53,0.70]	0.62 [0.53,0.72]	0.66 [0.59,0.74]
	T_2_w	0.74 [0.68,0.79]	0.73 [0.68,0.78]	0.73 [0.68,0.78]

The original approach involved pre-registration of inputs to high-field (HF) scans within participants, and erroneous use of only two planes (COR and SAG) as inputs (“HF, 2-plane”). We investigated the impact of including the third (AXI) plane, as originally intended (“HF, 3-plane”), and of pre-registering to a standard MNI template to avoid circularity (“MNI, 3-plane”). Values correspond to median [first, third quartile], i.e. Md [Q_1_, Q_3_], across 98 SynthSeg+ labels.

Results show that both the number of input scans (2 VS 3) and choice of pre-registration reference (individual 3 T HF scan VS MNI template) have a minimal impact on results. For most contrasts and statistics, median values across 98 SynthSeg+ labels are either identical, or only differ by 0.01-0.02. Only for Lin’s concordance correlation coefficient (CCC) is there a greater decrease in median value (up to 0.08).

As such, our corrected results remain consistent with our original conclusions, in that of the two contrasts (T_1_w and T_2_w) and five 64 mT scan resolutions investigated (AXI, COR, SAG, ISO, MRR), T_2_w MRR scans show optimal performance, independent of analytic decisions or errors within the MRR pipeline.

As previously, we note that both raw data and (now corrected) code are openly available, enabling replication or extension of our results.

