# Supplementary material for: Ultra-low-field brain MRI morphometry: Test–retest reliability and correspondence to high-field MRI
Source: Imaging Neurosci (Camb). 2025 Oct 15;3:IMAG.a.930. doi: 10.1162/IMAG.a.930 (PMC12529345; doi:10.1162/IMAG.a.930)
Supplement: Supplementary Material [file IMAG.a.930_supp.pdf]

Supplementary Information for: Ultra-low-field brain MRI morphometry: test-retest reliability and correspondence to high-field MRI

František Váša[*], Carly Bennallick, Niall J. Bourke, Francesco Padormo, Levente Baljer, Ula Briski, Paul Cawley, Tomoki Arichi, Tobias C. Wood, David J. Lythgoe, Flavio Dell'Acqua, Thomas C. Booth, Ashwin V. Venkataraman, Emil Ljungberg, Sean C.L. Deoni, Rosalyn J. Moran, Robert Leech[†], Steven C.R. Williams[†]

[*] Corresponding author. E-mail: frantisek.vasa@kcl.ac.uk.

[†] These authors contributed equally to this work.

List of Figures

S1 Participant demographics. 3
S2 Time between 64 mT scans, within participants. 3
S3 Differences in reliability and correspondence between small and large regions. 4
S4 Global reliability of **tissue volumes**, across contrasts and resolutions. 4
S5 Global reliability of **segmentations**, across contrasts and resolutions. 5
S6 Global correspondence to 3 T of **tissue volumes**, across contrasts and resolutions. . . 5
S7 Global correspondence to 3 T of **segmentations**, across contrasts and resolutions. . . 6
S8 All raw 64 mT scans in all three planes for one example participant. 6

List of Tables

S1 Acquisition parameters for 64 mT and 3 T scans. 7
S2 Percentage difference in tissue volumes, across contrasts and resolutions. 7
S3 Correspondence between local age-statistics, across contrasts and resolutions. 8
S4 Assignment of SynthSeg+ regions to tissue classes. 9
S5 Quality control of 64 mT registrations. 10
S6 Dice overlap for scans of good registration quality, across contrasts and resolutions. . . 10

Supplementary Text

Multi-Resolution Registration

We used multi-resolution registration (Deoni et al., 2022) to combine the three orthogonal non-isotropic 64 mT scans (AXI, COR, SAG) into a single "MRR" scan, of a higher effective resolution, using ANTs (Avants et al., 2009).

We first pre-aligned scans, and resampled them to $1{\times}1{\times}1$ mm^3, by rigid-registering them to the high-field scan of the corresponding contrast (i.e. T_2w or T_1w), using *antsRegistrationSyN.sh*.

We subsequently applied the *antsMultivariateTemplateConstruction2.sh* command, with the following options (based on Deoni et al., 2022):

- Transformation model: "SyN" (Greedy SyN)
- Similarity metric used for pairwise registration: "MI" (mutual information)
- Maximum iteration limit: 12

Other options were left unchanged from default values, including:

- Image statistic used to summarize images: mean of normalized intensities (As described in the aforementioned script: *"Normalization here means dividing each image by its mean intensity.")*
- Sharpening applied to template at each iteration: Laplacian
- Gradient step size: 0.25

For further details, see the accompanying code (the link is located within the main manuscript).

Supplementary Figures

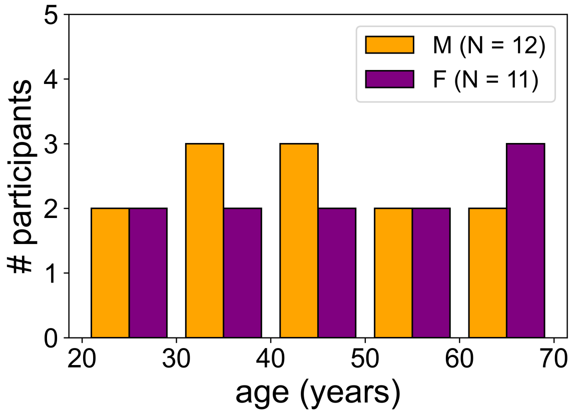

Figure S1: **Participant demographics.** Distribution of participants as a function of age, stratified by sex.

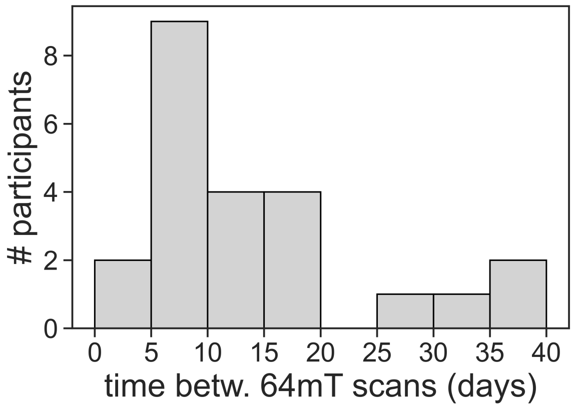

Figure S2: **Time between 64 mT scans, within participants.** The first 64 mT scan was acquired on the same day as, and immediately following, the 3 T scan.

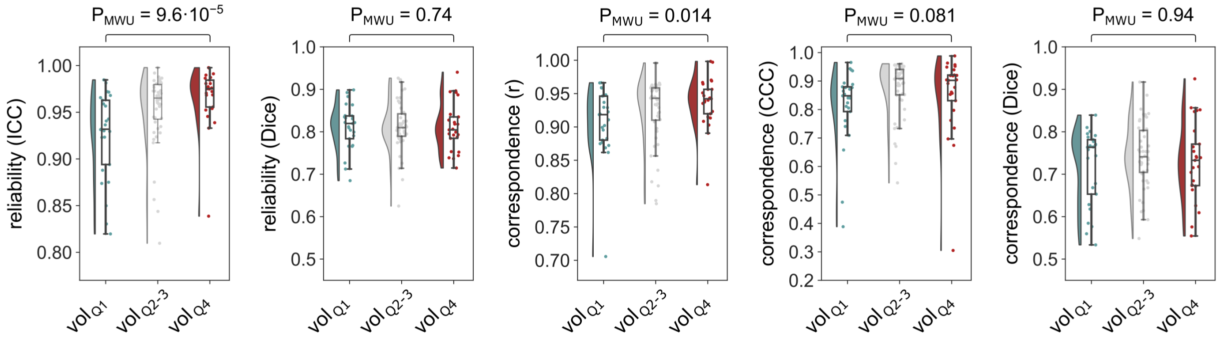

Figure S3: **Differences in reliability and correspondence between small and large regions.** The P-value (P_{MWU}) corresponds to a Mann-Whitney U test between the bottom and top quartile by average region volume (extracted from high-field scans), for each reliability and correspondence statistic. The middle two quartiles are included for illustration purposes.

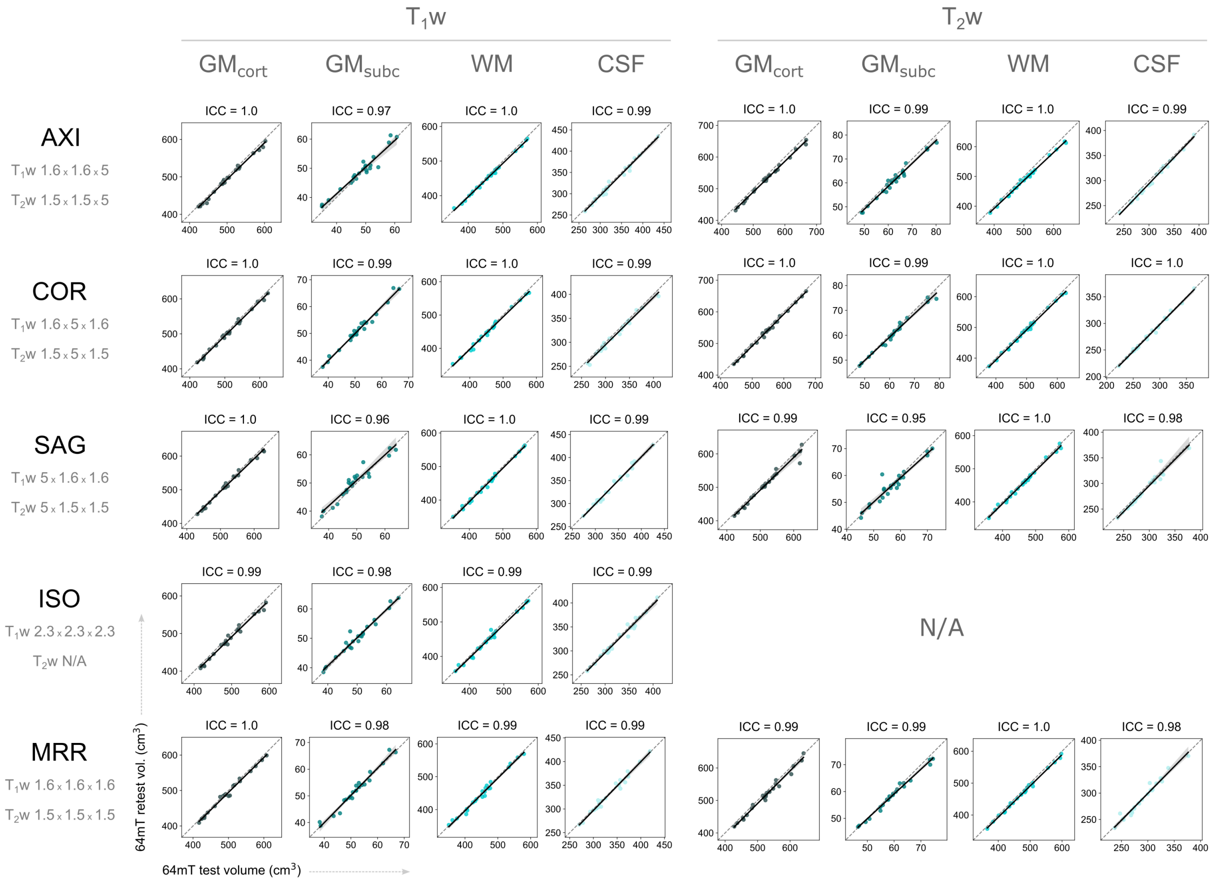

Figure S4: **Global reliability of 64 mT tissue volumes, across contrasts and resolutions.**

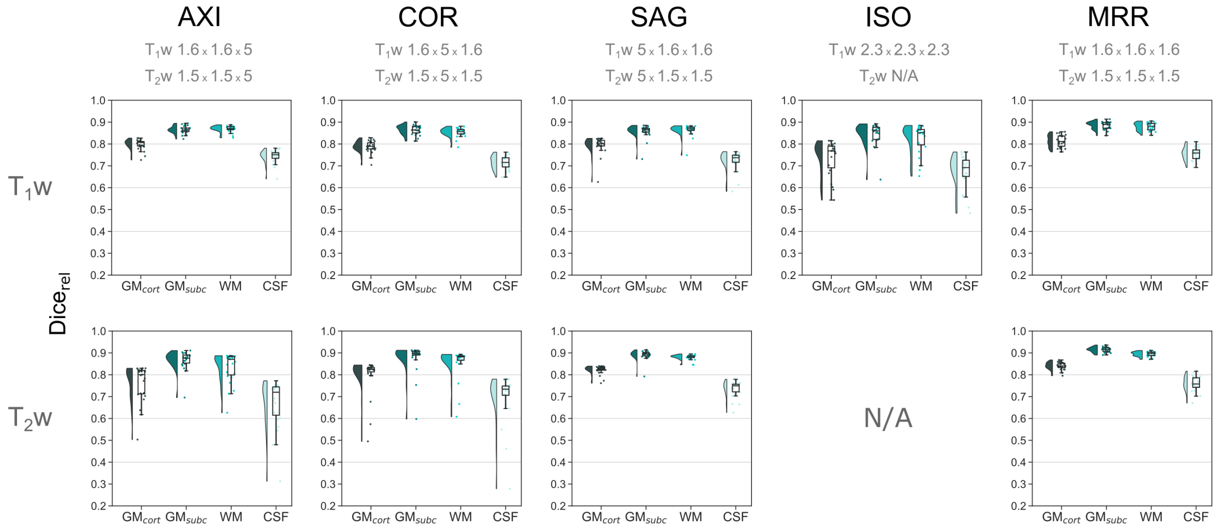

Figure S5: **Global reliability of 64 mT segmentations, across contrasts and resolutions.**

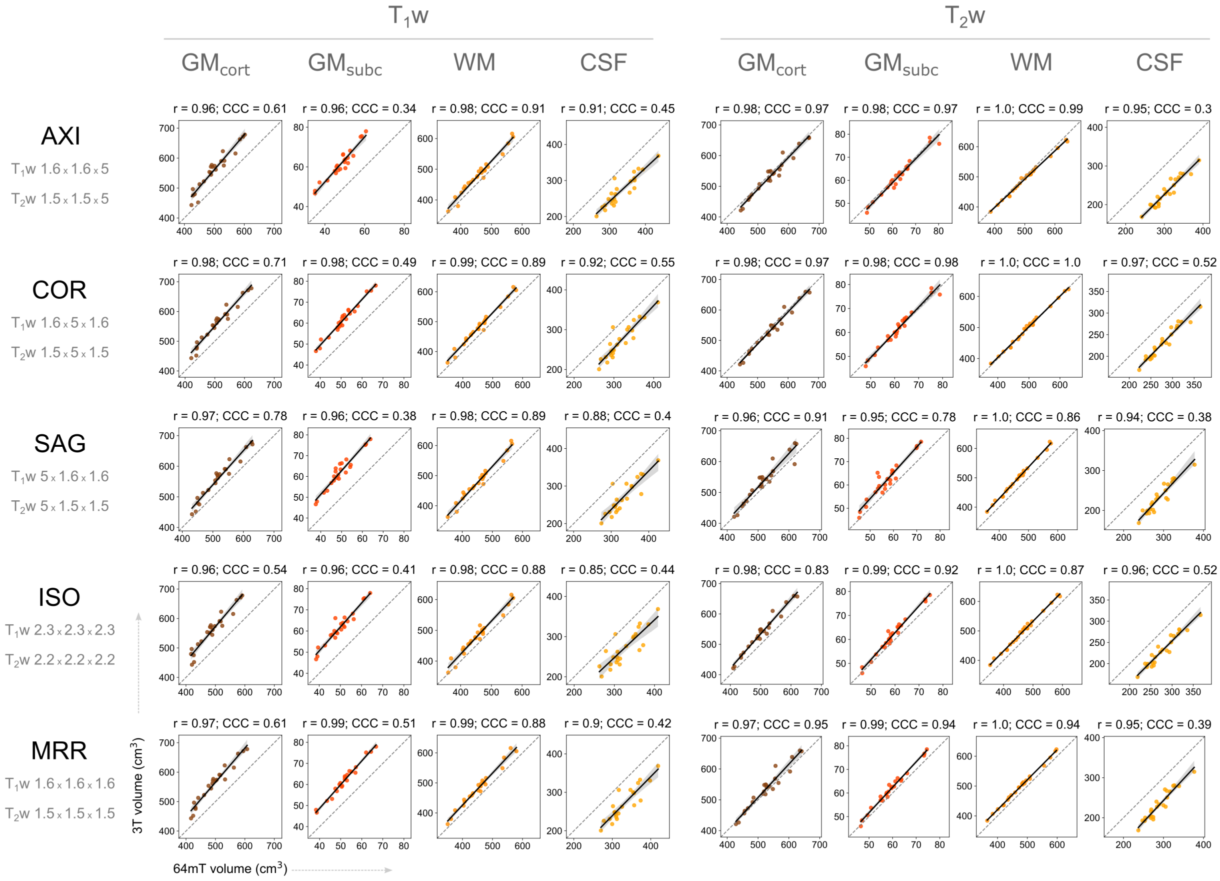

Figure S6: **Global correspondence of 64 mT tissue volumes to 3 T, across contrasts and resolutions.**

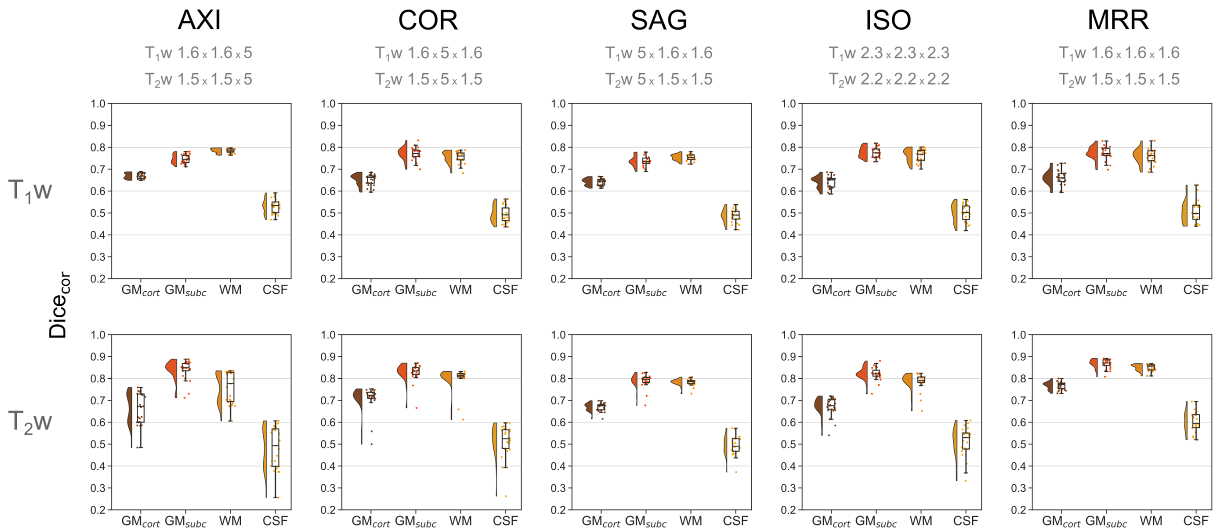

Figure S7: Global correspondence of 64 mT segmentations to 3 T, across contrasts and resolutions.

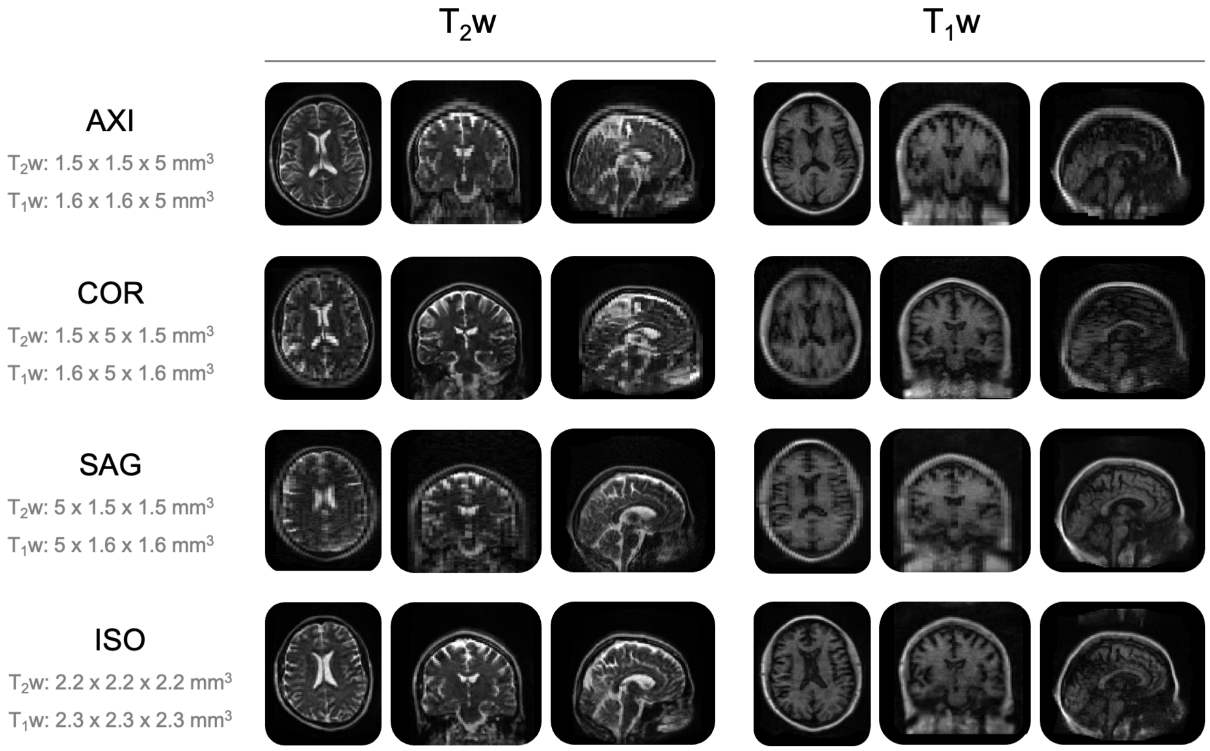

Figure S8: All raw 64 mT scans in all three planes for one example participant.

Supplementary Tables

Table S1: **Acquisition parameters for 64 mT and 3 T scans.** For all 64 mT scans, the FOV is 22×18×20 cm. *The MRR volume consists of a "multi-resolution registration" reconstruction (Deoni et al., 2022) of the AXI, COR and SAG volumes. †Accordingly, the MRR acquisition time corresponds to the sum of the acquisition times of the AXI, COR and SAG volumes (i.e. it does not require additional scan time beyond these three scans).

field	contrast	name	resolution (mm)	vox. vol. (mm^3)	matrix size	TR / TE / TI (s)	duration (min:s)
64 mT	T_1w	AXI	1.6×1.6×5	12.8	112×138×40	1.5 / 0.007 / 0.3	6:16
		COR	1.6×5×1.6	12.8	112×44×124	1.5 / 0.007 / 0.3	5:31
		SAG	5×1.6×1.6	12.8	36×138×125	1.5 / 0.007 / 0.3	6:13
		ISO	2.3×2.3×2.3	12.8	78×94×86	1.5 / 0.006 / 0.3	9:26
		MRR*	1.6×1.6×1.6	4.1	N/A	N/A	18:00†
	T_2w	AXI	1.5×1.5×5	11.3	120×146×40	2.0 / 0.17 / N/A	6:25
		COR	1.5×5×1.5	11.3	120×40×134	2.0 / 0.19 / N/A	5:21
		SAG	5×1.5×1.5	11.3	36×146×146	2.0 / 0.20 / N/A	5:49
		ISO	2.2×2.2×2.3	11.4	80×96×92	2.0 / 0.15 / N/A	9:49
		MRR*	1.5×1.5×1.5	3.4	N/A	N/A	17:35†
3 T	T_1w	MPRAGE	1.0×1.0×1.0	1.0	208×256×256	2.5 / 0.003 / 1.06	5:01
	T_2w	FSE	1.0×1.0×1.0	1.0	208×256×256	3.2 / 0.07 / N/A	6:06

Table S2: **Summary of percentage difference in tissue volumes from 64 mT scans relative to 3 T scans, across contrasts and resolutions.** Global values correspond to the median across 4 global tissue classes; local values correspond to the median, and first and third quartiles, across 98 structures segmented by SynthSeg+.

	contrast	MRR<br>T_1w 1.6×1.6×1.6<br>T_2w 1.5×1.5×1.5	ISO<br>T_1w 2.3×2.3×2.3<br>T_2w 2.2×2.2×2.2	AXI<br>T_1w 1.6×1.6×5<br>T_2w 1.5×1.5×5	COR<br>T_1w 1.6×5×1.6<br>T_2w 1.5×5×1.5	SAG<br>T_1w 5×1.6×1.6<br>T_2w 5×1.5×1.5
**Global** (Md)	T_1w	−9.3	−9.5	−8.2	−7.7	−7.0
	T_2w	−2.6	−5.3	+1.6	+0.8	−4.9
**Local** (Md [Q_1,Q_3])	T_1w	−11.3 [−15.4,−7.3]	−12.7 [−22.4,−7.4]	−12.3 [−23.1,−5.9]	−11.7 [−20.5,−4.0]	−10.1 [−21.5,−3.0]
	T_2w	−1.6 [−4.4,+0.9]	−3.8 [−11.0,−0.5]	+2.3 [−5.7,+7.1]	+2.6 [−6.4,+7.4]	−3.7 [−9.8,+1.6]

Table S3: Summary of correspondence between local age-statistics extracted from 64 mT and 3 T scans, across contrasts and resolutions. Linear models of regional tissue volume as a function of age and sex were used to extract t-statistics for the effect of age, and variance explained by age and sex (r^2), separately for 3 T and 64 mT scans. The correspondence of both effect sizes between field strengths, across regions was quantified using Pearson's r, and Lin's CCC, across both contrasts and all 64 mT scan resolutions.

effect size	corresp. statistic	contrast	MRR<br>T_1w 1.6×1.6×1.6<br>T_2w 1.5×1.5×1.5	ISO<br>T_1w 2.3×2.3×2.3<br>T_2w 2.2×2.2×2.2	AXI<br>T_1w 1.6×1.6×5<br>T_2w 1.5×1.5×5	COR<br>T_1w 1.6×5×1.6<br>T_2w 1.5×5×1.5	SAG<br>T_1w 5×1.6×1.6<br>T_2w 5×1.5×1.5
**t-stat. (age)**	**Pearson's r**	T_1w	0.83	0.78	0.79	0.82	0.77
		T_2w	0.90	0.84	0.83	0.84	0.77
	**Lin's CCC**	T_1w	0.70	0.61	0.60	0.68	0.53
		T_2w	0.74	0.70	0.67	0.70	0.54
$\mathbf{r^2}$	**Pearson's r**	T_1w	0.72	0.71	0.57	0.65	0.56
		T_2w	0.78	0.70	0.63	0.68	0.54
	**Lin's CCC**	T_1w	0.69	0.54	0.39	0.49	0.37
		T_2w	0.71	0.59	0.53	0.55	0.37

Table S4: **Assignment of SynthSeg+ regions to tissue classes.** The first column contains region names, as provided by SynthSeg+ (Billot et al., 2023). The second column contains the assignment of each region to one of four tissue classes; GM_{cort}: cortical gray matter, GM_{subc}: subcortical gray matter, WM: white matter, CSF: cerebrospinal fluid (cerebellar regions and the brainstem were not assigned to any of the four classes). The third colum contains the label of each region in the .nii.gz segmentation files generated by SynthSeg+.

name	tissue	label
Left-Cerebral-White-Matter	WM	2
Left-Lateral-Ventricle	CSF	4
Left-Inf-Lat-Vent	CSF	5
Left-Cerebellum-White-Matter	-	7
Left-Cerebellum-Cortex	-	8
Left-Thalamus-Proper	GM_{subc}	10
Left-Caudate	GM_{subc}	11
Left-Putamen	GM_{subc}	12
Left-Pallidum	GM_{subc}	13
3rd-Ventricle	CSF	14
4th-Ventricle	CSF	15
Brain-Stem	-	16
Left-Hippocampus	GM_{subc}	17
Left-Amygdala	GM_{subc}	18
CSF	CSF	24
Left-Accumbens-area	GM_{subc}	26
Left-VentralDC	GM_{subc}	28
Right-Cerebral-White-Matter	WM	41
Right-Lateral-Ventricle	CSF	43
Right-Inf-Lat-Vent	CSF	44
Right-Cerebellum-White-Matter	-	46
Right-Cerebellum-Cortex	-	47
Right-Thalamus-Proper	GM_{subc}	49
Right-Caudate	GM_{subc}	50
Right-Putamen	GM_{subc}	51
Right-Pallidum	GM_{subc}	52
Right-Hippocampus	GM_{subc}	53
Right-Amygdala	GM_{subc}	54
Right-Accumbens-area	GM_{subc}	58
Right-VentralDC	GM_{subc}	60
ctx-lh-bankssts	GM_{cort}	1001
ctx-lh-caudalanteriorcingulate	GM_{cort}	1002
ctx-lh-caudalmiddlefrontal	GM_{cort}	1003
ctx-lh-cuneus	GM_{cort}	1005
ctx-lh-entorhinal	GM_{cort}	1006
ctx-lh-fusiform	GM_{cort}	1007
ctx-lh-inferiorparietal	GM_{cort}	1008
ctx-lh-inferiortemporal	GM_{cort}	1009
ctx-lh-isthmuscingulate	GM_{cort}	1010
ctx-lh-lateraloccipital	GM_{cort}	1011
ctx-lh-lateralorbitofrontal	GM_{cort}	1012
ctx-lh-lingual	GM_{cort}	1013
ctx-lh-medialorbitofrontal	GM_{cort}	1014
ctx-lh-middletemporal	GM_{cort}	1015
ctx-lh-parahippocampal	GM_{cort}	1016
ctx-lh-paracentral	GM_{cort}	1017
ctx-lh-parsopercularis	GM_{cort}	1018
ctx-lh-parsorbitalis	GM_{cort}	1019
ctx-lh-parstriangularis	GM_{cort}	1020
⋮	⋮	⋮

name	tissue	label
⋮	⋮	⋮
ctx-lh-pericalcarine	GM_{cort}	1021
ctx-lh-postcentral	GM_{cort}	1022
ctx-lh-posteriorcingulate	GM_{cort}	1023
ctx-lh-precentral	GM_{cort}	1024
ctx-lh-precuneus	GM_{cort}	1025
ctx-lh-rostralanteriorcingulate	GM_{cort}	1026
ctx-lh-rostralmiddlefrontal	GM_{cort}	1027
ctx-lh-superiorfrontal	GM_{cort}	1028
ctx-lh-superiorparietal	GM_{cort}	1029
ctx-lh-superiortemporal	GM_{cort}	1030
ctx-lh-supramarginal	GM_{cort}	1031
ctx-lh-frontalpole	GM_{cort}	1032
ctx-lh-temporalpole	GM_{cort}	1033
ctx-lh-transversetemporal	GM_{cort}	1034
ctx-lh-insula	GM_{cort}	1035
ctx-rh-bankssts	GM_{cort}	2001
ctx-rh-caudalanteriorcingulate	GM_{cort}	2002
ctx-rh-caudalmiddlefrontal	GM_{cort}	2003
ctx-rh-cuneus	GM_{cort}	2005
ctx-rh-entorhinal	GM_{cort}	2006
ctx-rh-fusiform	GM_{cort}	2007
ctx-rh-inferiorparietal	GM_{cort}	2008
ctx-rh-inferiortemporal	GM_{cort}	2009
ctx-rh-isthmuscingulate	GM_{cort}	2010
ctx-rh-lateraloccipital	GM_{cort}	2011
ctx-rh-lateralorbitofrontal	GM_{cort}	2012
ctx-rh-lingual	GM_{cort}	2013
ctx-rh-medialorbitofrontal	GM_{cort}	2014
ctx-rh-middletemporal	GM_{cort}	2015
ctx-rh-parahippocampal	GM_{cort}	2016
ctx-rh-paracentral	GM_{cort}	2017
ctx-rh-parsopercularis	GM_{cort}	2018
ctx-rh-parsorbitalis	GM_{cort}	2019
ctx-rh-parstriangularis	GM_{cort}	2020
ctx-rh-pericalcarine	GM_{cort}	2021
ctx-rh-postcentral	GM_{cort}	2022
ctx-rh-posteriorcingulate	GM_{cort}	2023
ctx-rh-precentral	GM_{cort}	2024
ctx-rh-precuneus	GM_{cort}	2025
ctx-rh-rostralanteriorcingulate	GM_{cort}	2026
ctx-rh-rostralmiddlefrontal	GM_{cort}	2027
ctx-rh-superiorfrontal	GM_{cort}	2028
ctx-rh-superiorparietal	GM_{cort}	2029
ctx-rh-superiortemporal	GM_{cort}	2030
ctx-rh-supramarginal	GM_{cort}	2031
ctx-rh-frontalpole	GM_{cort}	2032
ctx-rh-temporalpole	GM_{cort}	2033
ctx-rh-transversetemporal	GM_{cort}	2034
ctx-rh-insula	GM_{cort}	2035

(N/A values are due to T_2w ISO scans not being acquired on the ENIC 64 mT scanner; see Methods.)

Table S5: **Quality control of 64 mT registrations.** Each ultra-low-field scan was rigid-registered to the high-field scan of the corresponding contrast, within participants, using FSL FLIRT (Jenkinson and Smith, 2001). The table contains the number of scans for each registration quality score (1 = perfect / 5 = fail), by ultra-low-field contrast and resolution. We note that readers can assess registration quality by themselves, using publicly available data shared alongside this manuscript, which includes registered scans and segmentations (upon publication).

			**score (1 = perfect / 5 = fail)**					
**scanner**	**contrast**	**resolution**	**1**	**2**	**3**	**4**	**5**	**OK** (≤ 3)
CNS	T_1w	AXI	11	7	3	2	0	21
		COR	3	8	6	5	1	17
		SAG	11	10	1	0	0	23
		ISO	3	10	3	4	3	16
		MRR	8	8	5	1	0	22
	T_2w	AXI	7	1	10	3	2	18
		COR	12	5	4	1	1	21
		SAG	4	12	4	2	1	20
		ISO	0	1	10	12	0	11
		MRR	12	9	1	1	0	22
ENIC	T_1w	AXI	11	7	3	2	0	21
		COR	8	9	4	2	0	21
		SAG	12	6	5	0	0	23
		ISO	4	6	6	4	3	16
		MRR	11	6	4	2	0	21
	T_2w	AXI	10	6	4	3	0	20
		COR	9	12	1	1	0	22
		SAG	2	15	4	2	0	21
		ISO	N/A	N/A	N/A	N/A	N/A	N/A
		MRR	8	15	0	0	0	23

Table S6: **Summary of Dice reliability and correspondence for scans of sufficient registration quality, across contrasts and resolutions.** Global values correspond to the median across 4 global tissue classes. Local values correspond to the median, and first and third quartiles, i.e. Md [Q_1, Q_3], across 98 SynthSeg+ labels. Values which show a (0.01) difference relative to the main results (with all scans included) are highlighted in bold. (N/A values are due to T_2w ISO scans being acquired on one 64 mT scanner only; see Methods.)

			**MRR**	**ISO**	**AXI**	**COR**	**SAG**
		**contrast**	T_1w 1.6×1.6×1.6	T_1w 2.3×2.3×2.3	T_1w 1.6×1.6×5	T_1w 1.6×5×1.6	T_1w 5×1.6×1.6
			T_2w 1.5×1.5×1.5	T_2w 2.2×2.2×2.2	T_2w 1.5×1.5×5	T_2w 1.5×5×1.5	T_2w 5×1.5×1.5
**Global**	**Dice$_{rel}$**	T_1w	0.85	0.81	0.84	**0.83**	0.83
		T_2w	0.87	N/A	0.84	0.85	0.86
	**Dice$_{cor}$**	T_1w	0.71	0.71	0.71	**0.72**	0.69
		T_2w	0.82	0.73	0.73	0.77	0.73
**Local**	**Dice$_{rel}$**	T_1w	0.79 [0.77,0.82]	**0.73** [**0.69**,0.77]	0.75 [0.73,0.79]	0.74 [**0.70**,0.77]	0.75 [**0.72**,0.78]
		T_2w	0.81 [**0.78**,0.84]	N/A	0.76 [0.71,0.79]	0.77 [0.74,0.81]	0.77 [0.75,0.81]
	**Dice$_{cor}$**	T_1w	0.61 [0.53,0.70]	0.59 [0.52,0.69]	0.61 [0.54,0.66]	**0.60** [**0.52**,0.69]	0.56 [**0.49**,**0.65**]
		T_2w	0.74 [0.68,0.79]	0.65 [0.57,0.72]	**0.61** [**0.51**,**0.70**]	0.67 [**0.61**,0.73]	0.59 [0.54,0.66]

Supplementary References

Billot, B., Magdamo, C., Cheng, Y., Arnold, S.E., Das, S., Iglesias, J.E.: Robust machine learning segmentation for large-scale analysis of heterogeneous clinical brain MRI datasets. Proceedings of the National Academy of Sciences **120**(9), 2216399120 (2023) https://doi.org/10.1073/pnas.2216399120 . Accessed 2023-08-22

Deoni, S.C.L., O'Muircheartaigh, J., Ljungberg, E., Huentelman, M., Williams, S.C.R.: Simultaneous high-resolution T2 -weighted imaging and quantitative T2 mapping at low magnetic field strengths using a multiple TE and multi-orientation acquisition approach. Magnetic Resonance in Medicine **88**(3), 1273–1281 (2022) https://doi.org/10.1002/mrm.29273

Jenkinson, M., Smith, S.: A global optimisation method for robust affine registration of brain images. Medical Image Analysis **5**(2), 143–156 (2001) https://doi.org/10.1016/S1361-8415(01)00036-6 . Accessed 2024-07-25
